# Strain-Controlled Recombination in InGaN/GaN Multiple Quantum Wells on Silicon Substrates

**DOI:** 10.1186/s11671-018-2663-6

**Published:** 2018-08-22

**Authors:** Tao Lin, Zhi Yan Zhou, Yao Min Huang, Kun Yang, Bai Jun Zhang, Zhe Chuan Feng

**Affiliations:** 1 School of Physical Science and Technology, Laboratory of Optoelectronic Materials and Detection Technology, Guangxi Key Laboratory for Relativistic Astrophysics,, Guangxi University, Nanning, 530004 China; 2 School of Electronics and Information Technology, State Key Laboratory of Optoelectronic Materials and Technologies, Sun Yat-Sen University, Guangzhou, 510275 China

**Keywords:** InGaN/GaN multiple quantum well, Luminescence, Time-resolved photoluminescence, Silicon substrate

## Abstract

This paper reports the photoluminescence (PL) properties of InGaN/GaN multiple quantum well (MQW) light-emitting diodes grown on silicon substrates which were designed with different tensile stress controlling architecture like periodic Si δ-doping to the n-type GaN layer or inserting InGaN/AlGaN layer for investigating the strain-controlled recombination mechanism in the system. PL results turned out that tensile stress released samples had better PL performances as their external quantum efficiencies increased to 17%, 7 times larger than the one of regular sample. Detail analysis confirmed they had smaller nonradiative recombination rates ((2.5~2.8)×10^−2^ s^−1^ compared to (3.6~4.7)× 10^−2^ s^−1^), which was associated with the better crystalline quality and absence of dislocations or cracks. Furthermore, their radiative recombination rates were found more stable and were much higher ((5.7~5.8) ×10^−3^ s^−1^ compared to [9~7] ×10^−4^ s^−1^) at room temperature. This was ascribed to the suppression of shallow localized states on MQW interfaces, leaving the deep radiative localization centers inside InGaN layers dominating the radiative recombination.

## Background

InGaN/GaN multiple quantum well (MQW) structures grown on silicon substrates instead on conventional sapphire have attracted growing attentions for their potential applications in low-cost solid-state lighting, panel display, and silicon photonics [1–5]. The critical obstacle in fabricating high-quality GaN film on Si is the thermal expansion mismatch (56%) between GaN and Si, which introduced large tensile stress and cracks to the grown GaN films [6–9]. Furthermore, a Si doped n-type GaN layer beneath MQW layers is necessary for light-emitting diodes (LEDs) or laser diodes (LDs). In these cases, additional tensile stress from Si doping will be brought in. In recent years, efforts have been made to overcome these difficulties via using intermediate layers with suitable compressive stress to counterbalance tensile stress [10–16], delta doping for strain relaxation [17, 18], or the lattice-matched buffer layer deposition [19, 20]. According to previous works [17], periodic Si δ-doping architecture of the n-type GaN layer could achieve smoother GaN layer with higher crystalline quality and lower crack density than on Si uniformly doped GaN. This was attributed to the reduction of tensile stress. Several works have been done for examining the surface morphology, dislocation density, and electrical properties of crystalline GaN/Si δ-doping GaN layers on either sapphire [21, 22] or silicon substrates [23]. Unfortunately, few of them directly investigated the luminescence properties of InGaN/GaN MQW structures on top of a Si δ-doping GaN layer and clarified the relationship between luminescence efficiency enhancement and strain release caused by the film structure improvements, which are critical to the device fabrication. It is also worth mentioning that, direct measuring strain or observing lattice distortions without breaking down the LED samples is difficult. Indirect methods are always applied to evaluate the internal strain. For instance, mechanical pressure was applied to modulate the internal strain, which led to the changes of piezoelectric field inside MQWs as well as the optoelectronic performances of LED devices [24–27]. In any of these cases, luminescence spectra measurements were found indispensable for exanimating the strain-related device performance.

Therefore, in this work, InGaN/GaN MQW LED structures were deposited on crystalline silicon substrates. Either Si uniformly doped GaN or periodic Si δ-doped GaN working as n-type GaN layer was grown for comparison. Furthermore, two more control samples based on Si uniformly doped n-type GaN layers, inserting by a thin layer of AlGaN or InGaN respectively, were also prepared for supporting the analysis of influence of compressive stress or tensile stress to the device performance, as AlGaN has smaller lattice constant than GaN, which will partially release tensile stress on the surface, as well as InGaN inserted layer will aggravate tensile stress on the contrary. Relative photoluminescence (PL) efficiencies and recombination lifetimes (rates) for each sample were extracted from temperature-varied steady-state (SS) PL spectra and time-resolved (TR) PL spectra and then systematically analyzed. The results turned out that tensile stress released samples had better PL performances as both the nonradiative recombination related to structure defects were suppression and radiative recombination are more connected to deep recombination states inside InGaN well layers, which led to radiative recombination that are more stable with temperature.

## Methods

**Fig. 1:**
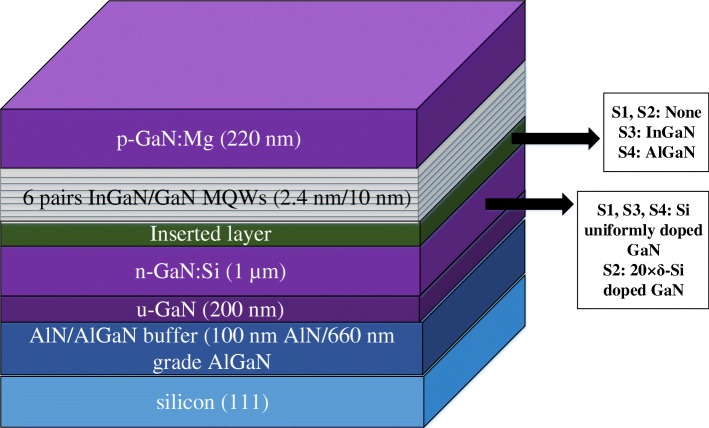
Structures of the MQW LED samples grown on Si substrates. S1, S3, and S4 contain 1 μm Si uniformly doped n-type GaN layer. S3 contains 20 nm InGaN inserted layer. S4 contains 20 nm AlGaN inserted layer. S2 contains 20 periods of Si-δ-doped planes each followed by 50 nm nominally undoped GaN with a total thickness of 1 μm instead of Si uniformly doped n-type GaN layer

As shown in the schematic of Fig. 1, the epitaxial growth of InGaN/GaN MQWs were performed by metal organic chemical vapor deposition which was reported in previous work [17]: 100 nm AlN layer, 660 nm linearly graded AlGaN layer, and 200 nm nominally undoped GaN layer were grown on the Si (111) substrate as the buffer at 1060, 1060, and 1020 °C, respectively. For samples S1, S3, and S4, 1 μm Si uniformly doped GaN layer was deposited on the buffer with the estimated Si atom concentration around 10^18^ cm^−3^. For samples S3 and S4, 20 nm InGaN inserted layer with In%~10at% or 20 nm AlGaN inserted layer with Al%~20% was deposited after the n-type Si uniformly doped GaN layer. For sample S2, 20 periods of Si δ-doped planes each followed by 50 nm nominally undoped GaN with a total thickness of 1 μm instead of Si uniformly doped GaN layer was grown on the buffer. After those, on each sample of S1–S4, 6 periods of InGaN/GaN QWs were grown at 800 °C, in which indium composition is around 22.0at%. The average well/barrier thickness was estimated as 2.4 nm/10 nm. After that, 220 nm Mg doped p-type GaN layers were grown at 1020 °C. For PL spectra tests, a Zolix-750 PL system with a 10 mW, 377 nm pulsed laser was used as the excitation light resource, and an ANDOR Newton CCD with 0.09 nm resolution was used as the photodetector. In TRPL measurements, the PL decays were recorded by a time-correlated single-photon counting system in 10–300 K.

## Results and Discussion

**Fig. 2:**
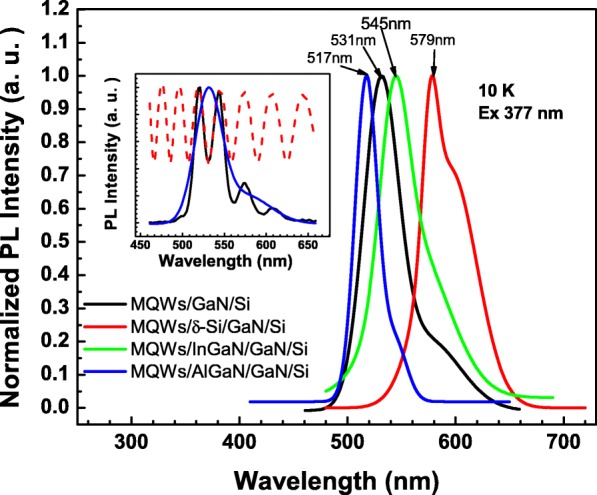
Overview of SSPL for S1–S4 excited by 377 nm laser at 10 K. The original PL signals contain Fabry-Perot oscillation which is shown as the black curve in the insert (S1 as an example). The oscillation item (red dash curve) and Gaussian PL peak (blue curve) are split by fitting the original signals. All PL data for S1–S4 are treated by the same method, and then, the split Gaussian PL are shown in the figure

The overview of SSPL properties for each tested MQW sample at 10 K is shown in Fig. 2. As seen in the insert, the original MQW on Si-structured S1 exhibits emission peaks around 500–650 nm with Fabry-Perot oscillation. PL spectra for all four samples have the same character. This phenomenon is commonly observed in GaN-based LED grown on Si substrates [28–30], as the buffer/Si interface has large reflectance, so a remarkable downward fraction of PL intensity from MQWs is reflected and interferes with the directly upward fraction. These oscillation peaks can be simply described as Gaussian PL signals multiplied by oscillation item (1 + Acos (4*πnd*/*λ*)) (demonstrated as the red curve in the insert of Fig. 1), in which *A* represents the oscillation strength, *n* is the average refractive index of MQW film, *d* is the whole thickness of MQW film, and *λ* is the PL wavelength. According to the above model, the original Gaussian PL peak can be fitted and extracted from the complex oscillation peaks (demonstrated as the blue curve in the insert of Fig. 1). The SSPL result turned out that S1 has a sharp green PL peak at 531 nm, according to the bandgap energy of InGaN crystal with In%~22at%. As comparisons, S2 with Si δ-doped n-type GaN layer has a noteworthily redshifted PL peak at 579 nm, S3 with InGaN inserted layer has a slightly blueshifted PL peak at 517 nm, and S4 with AlGaN inserted layer has a slightly redshifted PL peak at 545 nm. Considering that AlGaN inserted layer plays the role of releasing the tensile stress just familiar with the function of Si δ-doping, whereas InGaN inserted layer aggravates tensile stress, these results indicate that tensile stress on the substrate will lead to the blueshift of MQW PL position or enlargement of the average bandgap of InGaN well. The strain-release effect of Si δ-doped GaN layer is much stronger than that of introduction of inserted layer.

**Fig. 3:**
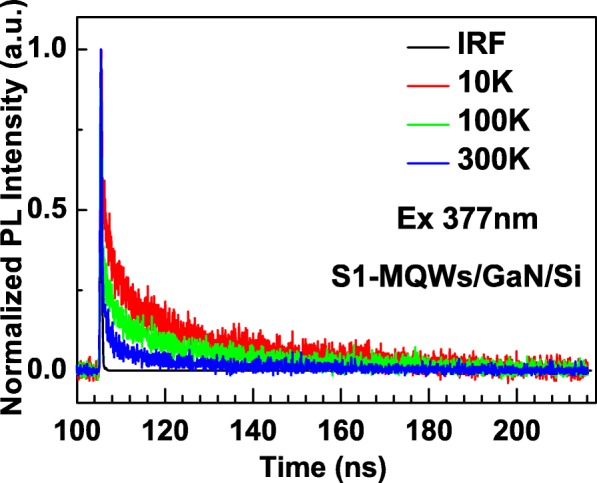
PL decay curves for S1 excited by 377 nm pulsed laser at 10 K, 100 K, and 300 K

**Fig. 4:**
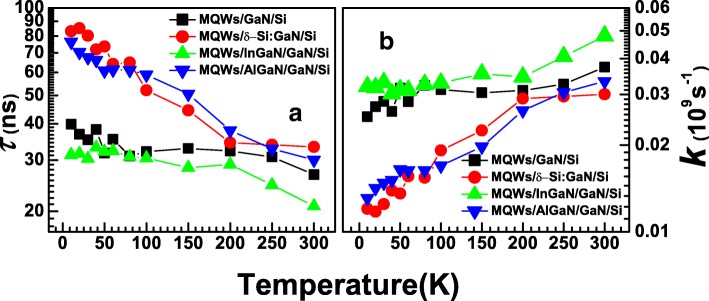
**a** PL lifetimes vs. temperature for S1–S4. The detected wavelength is kept at each peak position according to SSPL results in Fig. 2. **b** Corresponding recombination rates for S1–S4, which are obtained by *k* = 1/*τ*

For understanding the recombination nature in MQWs, it is critical to test their PL decay properties because PL lifetimes related to radiative/nonradiative recombination rates can be directly extracted from the decay curves. Here, the PL decays were measured with fixing the detected wavelength at peak values of S1–S4, and the measurements were done at different temperature ranged from 10 to 300 K. Figure 3 shows three typical PL decay curves for S1 tested at 10, 100, and 300 K. It is found that the PL decays for all S1–S4 tend to vary with temperature. This phenomenon reflects the temperature dependences of both radiative recombination rates and nonradiative recombination rates in the samples. Following single exponential decay function was used to fit every decay curve:

1 $$ I(t)={I}_0{e}^{-t/\tau } $$

where *I*_0_ represents the PL intensity at *t* = 0 and *τ* represents the PL lifetime. It is worth noted that not all decay curve can be perfectly fitted by above single exponential decay function. This has been widely discussed by several groups [31–34]. A reasonable assumption was that there exist multiple recombination centers in the system. Sometimes multi-exponential decay function was used to fit the curves. Here, to avoid introducing too many assumptions that are hard to be verified at last, or making the analysis incorrectly reflects only on the minor parts of the whole PL properties, we used the simplest model to extract an average PL lifetime for each sample, which may reflect the overall PL dynamic properties. The obtained lifetimes for S1–S4 were put together in Fig. 4a. To connect the PL dynamic results to the recombination probability, recombination rate *k* was defined as *k* = 1/*τ*. Spots of *k* versus temperature for S1–S4 are also shown in Fig. 4b. The results clearly show two diverse kinds of evolution of *k* with temperature that the recombination rates for the tensile stress released samples S2 and S4 are smaller than the one for original sample S1 or tensile stress aggravated sample S3 throughout the whole temperature range and increase more severely with increasing temperature. Note that *k* = *k*_*r*_ + *k*_*nr*_, in which *k*_*r*_ represents radiative recombination rate and *k*_*nr*_ represents nonradiative recombination rate. It is expected that *k*_*nr*_ increases when the temperature rises, and dominates *k* at room temperature, as it always relates to energy exchange processes with heat [35]. So, the *k* results at high temperature side in Fig. 4b exhibit the solid evidence that strain release processes such as Si δ-doping and AlGaN inserting have positive influences on suppressing nonradiative recombination in MQWs throughout reducing dislocation defects or cracks that have major influence on *k*_*nr*_. But *k*_*r*_ becomes nonnegligible on low temperature condition. Therefore, additional information and further analysis are needed to explain the behavior of *k* at low temperature side.

**Fig. 5:**
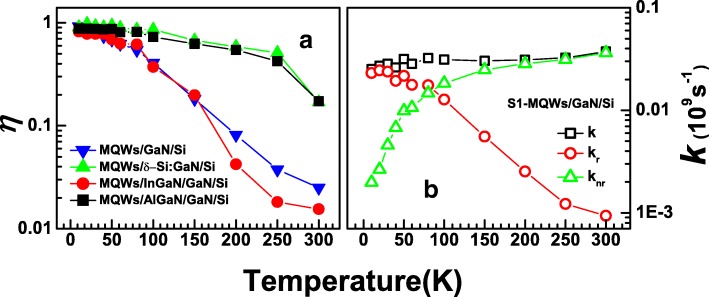
**a** Relative PL efficiencies vs. temperature for S1–S4. The detected wavelength is kept at each peak position according to SSPL results in Fig. 2. **b** Total recombination rate, radiative recombination rate, and nonradiative recombination rate vs. temperature for S1

Hence, for splitting *k*_*r*_ and *k*_*nr*_ from each *k* value, SSPL spectra on various temperature condition for each sample were measured. Then, the intensity of each PL peak corresponding to their detected wavelengths on previous TRPL tests were recorded as *I*(*T*). After that, relative PL efficiency was defined as *η* = *I*(*T*)/*I*_0_, in which *I*_0_ represents PL intensity at 0 K. The obtained PL efficiencies for S1–S4 were put together in Fig. 5a. It can be found that the PL efficiencies for S2 and S4 are both around 17%, which are 7 times larger than the one of S1. It is known that only radiative recombination contributes to PL intensity; therefore, this relative PL efficiency reflects the ratio of radiative recombination rate in total recombination rate:

2 $$ \eta ={k}_r/\left({k}_r+{k}_{nr}\right)={k}_r/k $$

**Fig. 6:**
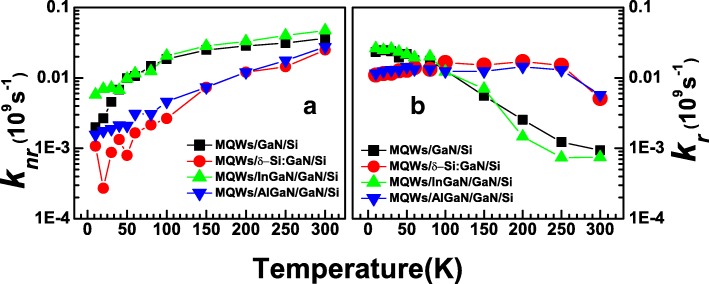
**a** Nonradiative recombination rates vs. temperature for S1–S4. **b** Radiative recombination rates vs. temperature for S1–S4

Thus, it is capable to resolve *k*_*r*_ = *kη* and *k*_*nr*_ = *k*(1 − *η*) from the TRPL results combined with *η*. The respective calculation for *k*_*r*_ and *k*_*nr*_ of S2 was shown in Fig. 5b as an example. The results turned out that even for S2 with Si δ-doping modification, nonradiative recombination rate is larger than radiative recombination rate until reaching a very low temperature of 50 K. This explains the reason why *k* keeps on increasing when temperature grows because it is dominant in *k*_*nr*_. It also indicates the high demand on further crystalline quality improvement for MQW on Si structures. The radiative recombination rate *k*_*r*_ was found declining monotonously with growing temperature, which does not agree with typical PL properties originating from free electron-hole pair recombination that *k*_*r*_ is free from temperature. However, it is reasonable if the PL process is dominant in exciton localization. Excitons tend to delocalize in higher temperature range; as a result, increasing of temperature will lead to decline of localization rate [32]. *k*_*nr*_ and *k*_*r*_ versus temperature for S1–S4 were summarized in Fig. 6a, b, respectively. As shown, the results of *k*_*nr*_ at 300 K for S2 and S4 are 2.5×10^−2^ s^−1^ and 2.8 ×10^−2^ s^−1^, respectively, which are lower than those for S1 (3.6 ×10^−2^ s^−1^) and S4 (4.7 ×10^−2^ s^−1^). These further verify that strain release processes suppress the formation of dislocation and cracks in MQWs, consequently decrease the densities of nonradiative recombination centers. This suppression effect becomes more sensitive when temperature goes down. The obtained *k*_*r*_ results are more complicated. As shown, *k*_*r*_ for S1 and S3 decline much more severely than that for S2 and S4 following temperature raise. As a result, obtained *k*_*r*_ at 300 K for S2 (5.7×10^−3^ s^−1^) and S4 (5.8 ×10^−3^ s^−1^) are much higher than that for S1 (9×10^−4^ s^−1^) and S3 (7 ×10^−4^ s^−1^). It is reasonable to ascribe this phenomenon to the strain release processes: according to the above discussion, the radiative processes in these MQW samples are mainly related to exciton recombination in localized states. Here, *k*_*r*_ is mainly determined by exciton localization rate *k*_loc_. The dramatical decline of *k*_loc_ with growing temperature indicates that the average depth of localized states is relatively small in the system, making the exciton easy to delocalize at high temperature. In another word, the average depths of localized states in samples with strain releasing as S1 and S3 are smaller than the ones without strain releasing. Based on the previous works [36], the localized radiative recombination centers in InGaN/GaN MQWs are often offered by structural defects in InGaN well layers, like well thickness variations and indium rich clusters, in which well thickness variations offer shallow states as well as indium rich clusters offer states with much deeper depths [33]. Here, the result of *k*_*r*_ indicates that strong tensile stress on MQW interfaces led by Si substrate and Si-doped GaN may improve the formation of radiative shallow structural defects, so the depth of localized states for S1 and S3 is smaller as well thickness variations are dominant in the exciton localization processes. For S2 and S4, the well thickness variations are suppressed, so the exciton localization processes are dominant in the deep states inside InGaN wells, exhibiting much larger average depths of localized states and more stable *k*_*r*_ versus temperature. Consequently, samples S1 and S3 demonstrate higher *k*_*r*_ than S2 and S4 at low temperature side where exciton delocalization effect is weak, but much smaller *k*_*r*_ at room temperature.

## Conclusions

In summary, temperature-varied SSPL and TRPL spectra were studied for different InGaN/GaN MQWs on Si structures with or without tensile stress releasing treatments. It was found that the sample with Si δ-doping GaN layer or AlGaN inserted layer had smaller recombination rate and higher PL efficiency (up to 17%) than the regular sample (2.5%) or sample with InGaN inserted layer (1.6%). Further analysis clarified that the smaller recombination rates were mainly led by smaller dominant nonradiative recombination rates (2.5 ×10^−2^ s^−1^ for δ-doping sample, 3.6 ×10^−2^ s^−1^ for AlGaN inserted sample compared to 3.6 ×10^−2^ s^−1^ for regular sample and 4.7 ×10^−2^ s^−1^ for InGaN inserted sample), which were ascribable to the suppression to the formation of dislocations or cracks. Besides smaller nonradiative recombination rates, the better PL performances were also led by the radiative recombination rates that were more stable and higher at room temperature (5.7 ×10^−3^ s^−1^ for δ-doping sample, 5.8 ×10^−3^ s^−1^ for AlGaN inserted sample compared to 9 ×10^−4^ s^−1^ for regular sample and 7 ×10^−4^ s^−1^ for InGaN inserted sample). They were also ascribable to the suppression of well thickness variations on MQW interfaces, leaving the deep radiative localization centers inside InGaN layers dominate the radiative recombination process. The above results showed a clear picture to the recombination processes of InGaN/GaN MQW LED devices on silicon substrates, which may guide the device fabrication in the future.

## Abbreviations

IQE: Internal quantum efficiency
LD: Laser diode
LED: Light-emitting diode
MQW: Multiple quantum well
PL: Photoluminescence
SSPL: Steady-state photoluminescence
TRPL: Time-resolved photoluminescence
